# Digitized and structured informed patient consent before contrast-enhanced computed tomography: feasibility and benefits in clinical routine

**DOI:** 10.1186/s13244-022-01304-6

**Published:** 2022-10-11

**Authors:** Markus Kopp, Jan Peter Roth, Frederik Geisler, Sascha Daniel, Theresa Ruettinger, Christoph Treutlein, Eva L. Balbach, Rafael Heiss, Matthias Wetzl, Nouhayla El Amrani, Alexander Cavallaro, Michael Uder, Matthias S. May

**Affiliations:** 1 Departement of Radiology, University Hospital Erlangen, Friedrich-Alexander-University (FAU) Erlangen-Nuremberg, 91054 Erlangen, Germany; 2 Imaging Science Institute, University Hospital Erlangen, Erlangen, Germany; 3 Friedrich-Alexander University Erlangen-Nuremberg, Erlangen, Germany; 4 Grenoble Alpes University, Grenoble, France

**Keywords:** Informed consent document, Personalized medicine, Helical computed tomography, Greenhouse gases, Electronic medical records

## Abstract

**Background:**

To evaluate the feasibility and benefits of digitized informed patient consent (D-IPC) for contrast-enhanced CT and compare digitized documentation with paper-based, conventional patient records (C-PR).

**Methods:**

We offered D-IPC to 2016 patients scheduled for a CT. We assessed patient history (e.g., CT examinations, malignant or cardiovascular diseases) and contraindications (red flags) for a CT (e.g., thyroid hyperfunction, allergies) using a tablet device. We evaluated the success rate of D-IPC and compared patient age between the subgroups of patients who were able or unable to complete D-IPC. We analyzed the prevalence of marked questions and red flags (RF). RF were compared with the documentation from C-PR. We estimated greenhouse gas (GHG) emissions for paperless workflow and provide a cost–benefit analysis.

**Results:**

Overall, 84.4% of patients completed D-IPC. They were younger (median 61 years) than unsuccessful patients (65 years; *p* < 0.001). Patients who marked questions (21.7%) were older than patients without inquiries (median 63.9 vs 59.5 years; *p* < 0.001). The most prevalent RF was thyroid disease (23.8%). RF were considered critical for contrast-agent injection in 13.7%, requiring personalized preparation. The detection rate for RF documented with D-IPC was higher than for C-PR (*n* = 385 vs. 43). GHG emissions for tablet production are 80–90 times higher than for paper production. The estimated costs were slightly higher for D-IPC (+ 8.7%).

**Conclusion:**

D-IPC is feasible, but patient age is a relevant factor. Marked questions and RF help personalize IPC. The availability of patient history by D-IPC was superior compared to C-PR.

## Key points

- D-IPC is feasible in more than 80% of our patients but higher patient age is associated with more difficulties in completing a D-IPC.
- Structured presentation of red flags and marked questions supports efficient and personalized patient–physician interaction.
- Paperless workflow reduces paper consumption and paper-related greenhouse gas emissions. However, estimated CO_2eq_ costs of tablet production are much higher.
- D-IPC has 8.7% higher operational costs, but the increased availability of structured clinical information has the potential to improve patient safety significantly.

## Background

Informed patient consent (IPC) is an essential component of patient care in many healthcare systems before contrast-enhanced CT [1–4]. The German Medical Association's professional code of conduct and the German Civil Code regulate that patients must be informed about medical procedures and medication applications [5, 6]. The justifying CT indication relies on the information provided by the patients and referring physicians. Severe adverse reactions may occur in contrast-enhanced studies. The European Society of Urogenital Radiology (ESUR) defined several critical issues for contrast-enhanced CT in their *Contrast Media Safety Guidelines* version 10.0 [7, 8]. Their structured evaluation during the IPC helps reduce patient risk to a minimum. Currently, conventional IPC (C-IPC) is mainly obtained and archived in hardcopy form. Such information is often hard to read, imprecise, and the included questionnaires are often incomplete. Vogele et al. [9] reported that more than 50% of the comments on a conventional IPC form are unreadable. These issues may lead to severe disturbances in the clinical workflow and potentially endanger patients' safety.

Moreover, hygiene aspects are critical. The disinfection of paper is limited, and both the staff and the patient often use the same pencil. After completing the exam, archiving of hard copies is done in binders or folders, hampering the ex-post access for further usage. Also, IPC retrieval in case of inquiries requires additional time effort of staff members under high time pressure and is error-prone. Moreover, storage of folders with C-IPC can be costly and seizes room capacity in the hospital. Structured, digitized access to the most relevant data could increase the efficiency and quality of the reporting process. Fully digitized patient self-assessment of informed patient consent (D-IPC) could help to increase the information available before and after the examination, reduce errors, and improve hygiene.

Several studies proved advantages of digitized patient history. Benaroia et al. found that patients well-accepted a history-taking computer device in an emergency department and that digitized patient triage did not delay patient care [10]. Kripalani et al. [11] found that tablet-based medication history in an emergency department is feasible and leads to more medication list updates. Lastly, Schlechtweg et al. reported already 2013 and 2014, 3–4 years after the iPad was released, that half the study patients who performed an iPad-based patient briefing before MRI preferred such workflows in the future. In contrast to other studies, Schlechtweg et al. showed that digital consent required more time to complete than handwritten consent [12, 13]. Despite partly conflicting results regarding the time efficiency of digitized patient history, these studies indicate that such systems could be helpful in radiology departments. However, it is still unproven if D-IPC with up-to-date software and user interface (compared to the almost 10-years-old study from Schlechtweg et al.) is feasibly in a high-performance academic center with quick CT turnover times due to high time pressure. Also, the higher-than-average age of a patient collective in an academic radiology department is potentially influencing the feasibility of D-IPC in a routine setting.

Previous studies analyzed the effect of digital reading methods on greenhouse gas (GHG) emissions compared to paper-reading and provide conflicting results concerning the environmental pollution [14, 15]. Scientific information about the GHG effects of paperless IPC is unavailable in the literature. In the radiological societies, GHG emissions of digitized workflow are still mainly disregarded. We compared saved GHG due to reduced paper consumption with the CO_2eq_ emissions caused by the tablet production.

The purpose of this investigation is to evaluate the feasibility and benefits of structured D-IPC for contrast-enhanced CT. Especially, we evaluate the null hypothesis that age does not influence the success rate and that D-IPC documentation is not superior compared to C-IPC.

## Methods

### Patient client

**Fig. 1:**
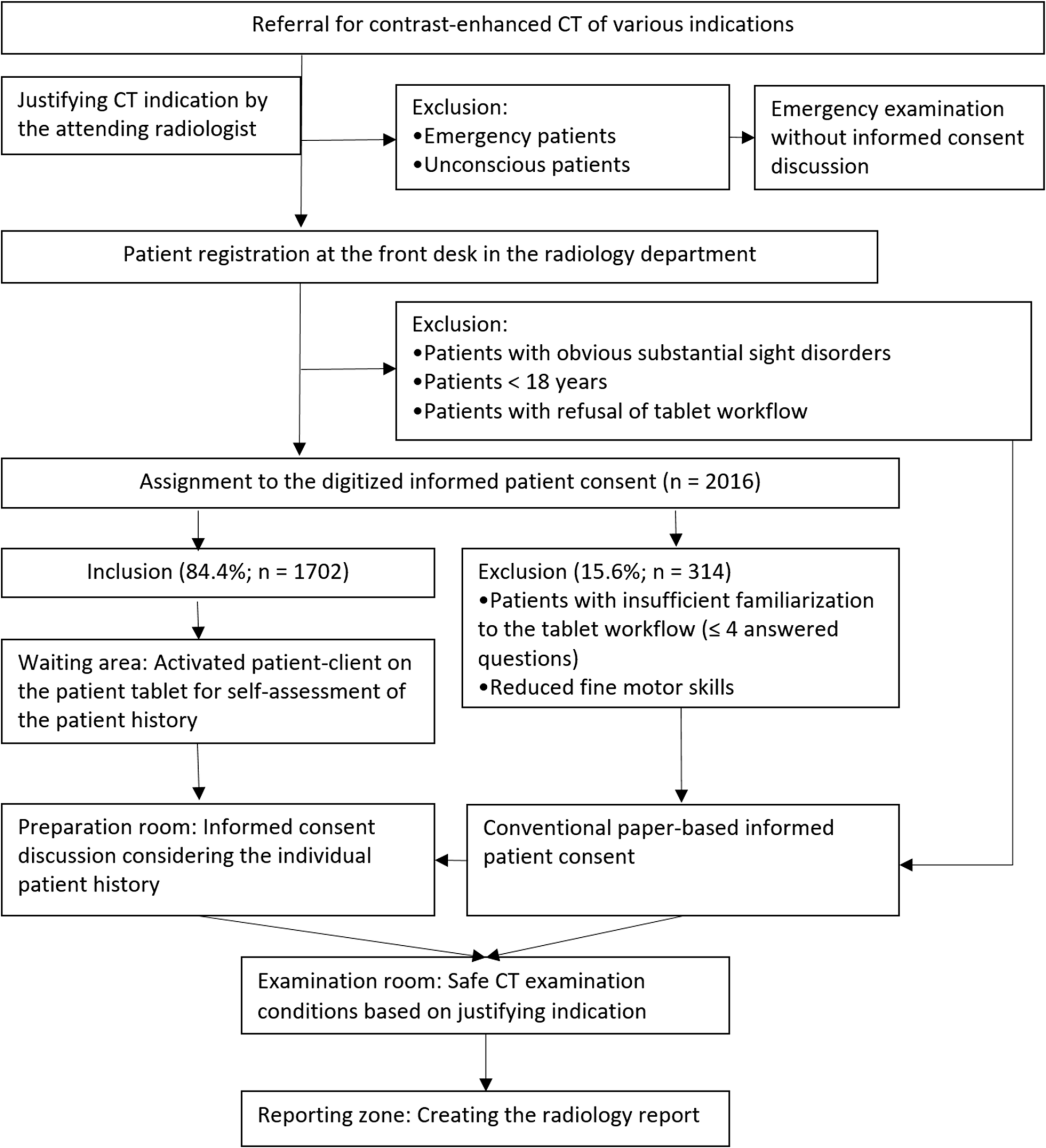
Study flowchart for tablet-based, digitized informed patient consent (D-IPC). Before the tablet-based workflow started, the indication for CT was evaluated by the attending physician. The patients were then asked to answer a dedicated questionnaire in the waiting room with 22 main items. Structured patient data based on D-IPC was available on all workstations for informed consent discussion and reporting process

We offered D-IPC to 2016 consecutive patients scheduled for a contrast-enhanced CT during a study period of 20 months (June 2018–February 2020). All examinations were carried out in a large university hospital performing around 34,000 CT examinations per year. Pediatric patients, emergency patients, patients with severe sight disorders, and unconscious patients were generally not considered study participants (Fig. 1). In addition, patients who refused to perform mobile tablet workflow were not assigned to D-IPC.

Before assigning patients to D-IPC, the attending radiologist evaluated the justifying CT indication based on the information provided by the referring physician and the already-archived information in the radiology information system (RIS) and the hospital information system. Patient master data were automatically transferred from the RIS worklist. The front desk in the radiology department allocated the contrast-enhanced CT questionnaires to the present patients using a desktop application of the D-IPC software (MEDePORT, Thieme Compliance GmbH, Erlangen, Germany). Patients were registered by manually typing the patient name and birthdate or in most cases by scanning a barcode from the web application on the desktop computer. The touch screen interaction was made available on a fleet of 10 mobile tablet devices (Surface Go, first-generation model, Microsoft Co., Redmond, WA, USA) using a dedicated patient-client application of the D-IPC software operating in the kiosk mode. We briefly introduced each patient, who stated sufficient German language skills, to the tablet functionality before starting the questionnaire with 22 main items in the waiting room without further assistance (e.g., by family members). The questionnaire consisted of items considering potential contraindication for contrast-enhanced CT (*n* = 7) and items about general patient history (*n* = 15). Altogether, the 22 main items are contributing to justify a safe CT indication (Tables 1, 2). The patients voluntarily answered each question. There was no constraint in answering every question. The attending radiologist discussed unanswered, critical questions during the personal informed patient discussion.

**Table 1:** General patient history. These items support the evaluation of the justifying CT indication with contrast agent injection, can help adapt the examination protocol and add information to the reporting process

General patient history
1. Age
2. Gender
3. Bodyweight
4. Body height
5. Prior computed tomography
6. Prior application of iodine-containing contrast agents
7. Anticoagulant medication
8. Metal implants
9. Cardiovascular diseases
10. Pulmonary or airway disease
11. Malignant disease
12. Metabolic disease
13. Liver disease
14. Gastrointestinal disease
15. Current or prior chemotherapy

**Table 2:** Red flags. We considered the following items about relative and absolute contraindications as highly relevant for justifying CT indication and protocol selection. A structured and easily accessible summary of red flag information was presented on the physician client before the informed consent discussion in the preparation room started

Relative and absolute contraindications
16. Prior complications after administration of iodine-containing contrast-agent
Allergic reactions against iodine-containing contrast-agents
Severe allergic reactions
17. Diabetes medication
Metformin containing drugs
18. Thyroid disease
Thyroid hyperfunction
19. Kidney disease
Reduced kidney function
Dialysis
20. Infectious disease
AIDS
Hepatitis
Tuberculosis
21. Pregnancy
22. Claustrophobia

The first four general questionnaire items consider patient gender, age, body weight, and height (Table 1) but do not consider specific patient history or contraindications for CT. Clinically relevant issues for justifying CT indication (red flags; Table 2) are only covered with five or more answered questionnaire items. Therefore, patients with four or fewer answered questions were excluded from the study after being assigned to D-IPC. All excluded patients underwent assisted C-IPC. We compared the age between the study patients and patients, who were unable to finalize D-IPC after already being assigned.

In addition to general patient history (Table 1), critical questions concerning relative and absolute contraindications were defined as red flags (Table 2). The patient-client functionality also allows the patients to mark unclear questions for personalized discussion with the physician.

After completion, the tablet devices were returned to the radiographer or the front desk, and the tablet cover and screen were immediately disinfected with dedicated wipes (Cleanisept® wipes forte, Dr.Schumacher, Malsfeld, Germany). The patients were then sent to the preparation room for the patient–physician discussion.

### Physician client

**Fig. 2:**
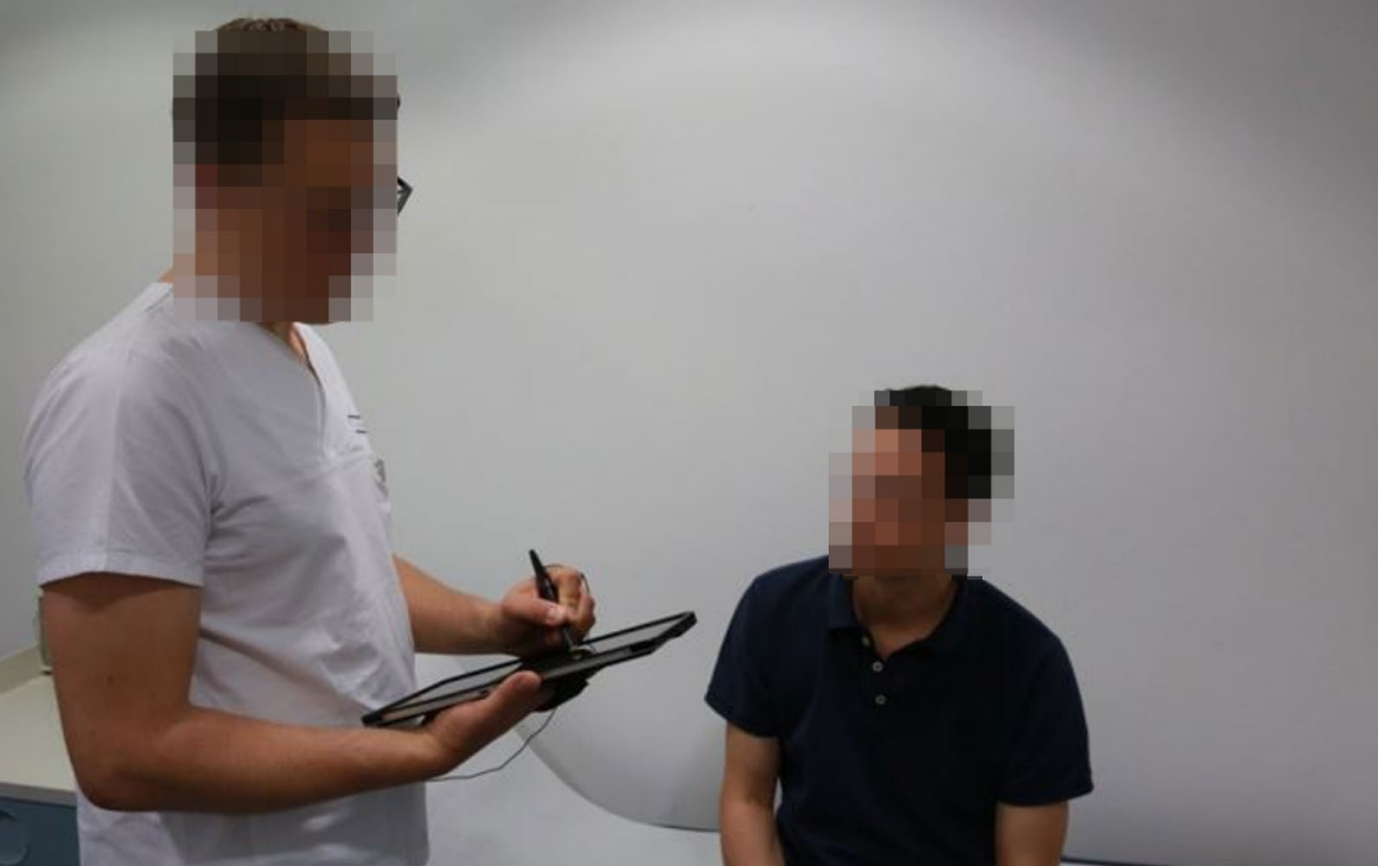
Digital signature of structured informed patient consent. Each patient and the attending physician digitally signed and confirmed the completeness and validity of the patient data in the preparation room

The on-premises software in the German language was installed on a local virtual server, and all data were stored in a local database. The attending radiologist used a dedicated physician client on two mobile tablet computers with a digital pen (Surface Pro 5, Microsoft Co., Redmont, WA, USA; Fig. 2) to review the questionnaires. Updates of the patient worklist were received automatically every 30 s from the RIS. Predefined filters are available at the physician's discretion: Red flags, individually marked questions, questions answered with yes, questions answered with no, and all questions. Red flags are always displayed first. Therefore, the informed consent discussion starts with a structured overview of potential contraindications. An illustration of the user interface is available in the literature [16]. Based on D-IPC recordings, a PDF document is generated and signed on the physician's tablet by the physician and the patient. Changes in content are impossible after the PDF is signed. The finalized PDF is automatically transferred to the enterprise picture archiving and communicating system (ePACS, synedra View Diagnostic, synedra information technologies GmbH, Innsbruck, Austria). Full access to the physician client, including all recorded data and filters, was available on all reporting stations. As the D-IPC software did not provide time stamps, we cannot provide precise measurements for IPC duration. However, for the cost–benefit analysis, we estimate the mean D-IPC and C-IPC duration to be approximately five minutes.

### Sustainability

The carbon dioxide (CO_2_) footprint for office paper sheets is reported in the literature as CO_2_ equivalents per sheet of paper with values of 4.29–4.74 g CO_2eq_ [17]. Each avoided conventional IPC consisted of two sheets of office paper. Based on these assumptions, we calculated reduced CO_2eq_ emissions achieved by paperless D-IPC. On the other hand, we also calculated the energy consumption and energy costs of the tablet devices (Table 5). Also, the tablet vendor provides estimated CO_2eq_ emissions for the production process of Surface Pro (121 kg CO_2eq_) and the Surface Go devices (107 kg CO_2eq_) [18].

### Cost–benefit analysis

We calculated a dedicated cost–benefit analysis (CBA) for D-IPC versus C-IPC for a 5 years project duration. For this project, we assumed a constant amount of 2016 informed patient consent forms for each year (Table 5). Our approach differentiates (1) personnel, (2) IT service, (3) hardware and (4) other operational costs. In addition, we compare the performance features of D-IPC and C-IPC for quality of care and patient safety.

We estimated an average of 5 min for our assistant staff to prepare D-IPC or C-IPC and to archive C-IPC. Moreover, we estimated the average time effort for the patient–physician discussion to be 5 min. The prevalence of extensive search procedures for IPC retrieval and consecutive CT vacancy is assumed 1%. We interviewed the technicians and the support staff about the average time effort of the search process. An average time effort of 10 min was specified. All IT service costs are specified by the vendor and the local IT service department. We used current wholesale pricing in Europe to estimate hardware costs for the tablets (Surface Pro; *n* = 2; Surface Go; *n* = 10). Energy consumption measurements of the physician`s tablet are provided by the vendor [18]. For each device, the estimated service duration per day was seven working hours, which results in an average energy cost of 5.3 Euro (31 Euro cents/kilowatt-hour). Paper costs were 1 cent for each sheet of paper based on current wholesale pricing in Europe. Printing costs were 7 cents per colored page. Each conventional informed consent form consists of two sheets of paper (4032 sheets). Professional disposal of patient data is crucial. However, disposable in Germany is allowed after 30 years of data storage. Therefore, this matter of expense does not apply to this study. We analyzed D-IPC and C-IPC workflow to identify important performance features.

### Database

The vendor of the D-IPC software provided a customized script for structured database export as an extensible markup language file (.XML). We processed this data with the MS Excel 2016 software package (Microsoft Cooperation, Redmond, WA, USA) and analyzed the prevalence of red flags, marked questions, and the rate of answered questionnaire items. Also, we compared for each patient the prevalence of documented red flags in C-PR and D-IPC. The information documented with C-PR included data from previously collected analog informed consents, the written requests from the referring physicians, and the documented risk factors and complications from previous examinations, each directly documented in the RIS. The information archived during D-IPC for the same patient was retrieved from the.XML file. We also evaluated the completeness of clinical context information and the examination-related clinical questions for each CT.

### Statistics

If applicable, we provide mean and standard deviation in the case of normal distribution. Median and interquartile range (IQR) with 75th and 25th percentiles are given when normal distribution was not assumed. Total patient counts for several questionnaire items do not sum to the same number for every category because multiple answers for pre-existing conditions or medications were allowed for most items. Moreover, there was no constraint in answering every questionnaire item. Normally distributed data were compared with paired t-tests. The Wilcoxon signed-rank test was used for further analysis if the normal distribution was not assumed. The significance level was defined as *p* < 0.05. We performed statistical analysis with the software package SPSS Statistics Version 21 (International Business Machines Corporation [IBM], Somers, NY, USA).

The study complied with the Declaration of Helsinki and was approved by the local institutional review board.

## Results

### Patient client

We included 2016 consecutive patients for this D-IPC study. A total of 314 (15.6%) patients were excluded after they had already been assigned to D-IPC due to incomplete questionnaires. These patients were significantly older (median 65 (IQR 57–74) years) than our 1702 included study patients (61 (IQR 52–68) years; *p* < 0.001). The rate of answered main questionnaire items was high (median 21 (IQR 21–21) questions). Most patients (88%, *n* = 1497) answered 90% or more of the 22 main items. In both groups, most patients were men (excluded: 60.5%; included: 62%). Under the included patients, the topics with the highest missing rate of response were the prior application of iodine-containing contrast agents (7.6%), malignant diseases (6.8%), and gastrointestinal diseases (5.3%, Table 3). The missing rate was below 5% for all red flag items (Table 4): Complications after contrast-agent application (2%), diabetes medication (3.3%), thyroid disease (3.9%), kidney disease (4,8%), infectious diseases (2.6%), pregnancy (0%) and claustrophobia (3.2%).

**Table 3:** Results for the general patient history based on digitized informed patient consent

General patient history (*n* = 1702)		Missing answers % (*n*)
1. Age	61 (IQR 52–68) years	0 (0)
2. Gender	♀: 38% (653)	0.4 (7)
	♂: 62% (1049)	
3. Body weight	77.4 ± 18.1 kg	2.4 (41)
4. Body height	170.4 ± 19 cm	3.7 (63)

	% (*n*)	
5. Prior computed tomography	87 (1481)	3.7 (63)
6. Prior application of iodine-containing contrast agents	86 (1463)	7.6 (130)
7. Anticoagulant medication	24.6 (419)	4.6 (78)
8. Metal implants	19.7 (336)	3.7 (63)
9. Cardiovascular diseases	41.6 (708)	5.3 (90)
Arterial hypertension	23.3 (397)	
Cardiac arrhythmia	7.1 (120)	
Shortness of breath	6.5 (111)	
Myocardial infarction	3.6 (62)	
Cerebral stroke	3.1 (53)	
Venous thrombosis	3.1 (53)	
Valvular heart disease	1.2 (21)	
Angina pectoris	0.8 (14)	
Aortic aneurysm	1.3 (24)	
Patient did not specify		2.5 (43)
10. Pulmonary or airway disease	22.9 (389)	5.3 (90)
Chronic obstructive pulmonary disease	7.8 (134)	
Asthma	4.3 (73)	
Sleep apnea	3.4 (58)	
Infectious pulmonary disease	1.4 (24)	
Emphysema	1.0 (17)	
Patient did not specify		8.3 (142)
11. Malignant disease	66.9 (1138)	6.8 (115)
12. Metabolic disease	14.6 (248)	2.4 (40)
13. Liver disease	13.8 (235)	4.8 (82)
14. Gastrointestinal disease	22.6 (385)	5.3 (90)
15. Current or prior chemotherapy	51.6 (878)	3.6 (62)

Missing answers are calculated concerning the whole study collective (*n* = 1702). Multiple selections of pre-existing conditions or medications were allowed. There was no constraint in answering each questionnaire item. The radiologist in charge discussed missing critical questionnaire items in the personal informed consent discussion and added documentation manually in the Portable Document Format (PDF)

**Table 4:** Prevalence of red flags about relative and absolute contraindications for contrast-enhanced computed tomography

Red flags	% (*n*)	Missing answer % (*n*)
16. Prior complications after administration of iodine-containing contrast-agent	5.8 (98)	2.0 (33)
Allergic reactions against iodine-containing contrast-agents	4.9 (84)	
Severe allergic reactions	0.3 (5)	
Vomiting and Nausea	0.8 (14)	
17. Diabetes medication	9.6 (164)	3.3 (56)
Metformin-containing drugs	0.5 (9)	
Metformin-containing drugs and reduced kidney function	0.06 (1)	
Insulin medication	4.8 (81)	
Oral medication other than Metformin	4.1 (69)	
Patient did not specify		1.0 (16)
18. Thyroid disease	23.8 (405)	3.9 (66)
Thyroid hypofunction	11.9 (203)	
Thyroid hyperfunction	2.0 (34)	
Goiter	3.5 (61)	
Thyroid surgery	4.5 (77)	
Thyroid cancer	0.8 (12)	
Hot thyroid nodule	0.4 (6)	
Basedow’s disease	0.4 (6)	
Patient did not specify		3.5 (60)
19. Kidney disease	12.5 (214)	4.8 (81)
Reduced kidney function	3.6 (62)	
Kidney surgery	2.6 (44)	
Kidney stones	1.8 (30)	
Hemodialysis	0.6 (11)	
Infection	0.3 (5)	
Hematuria	0.7 (12)	
Patient did not specify		4.0 (68)
20. Infectious disease	2.8 (48)	2.6 (44)
AIDS	0.4 (6)	
Hepatitis	1.5 (26)	
Tuberculosis	0.4 (7)	
Patient did not specify		0.5 (9)
21. Pregnancy	0.2 (3)*	(0) (0)
Unsure if pregnant	0.2 (3)	
22. Claustrophobia	9.7% (166)	3.2 (54)

Missing answers are calculated concerning the whole study collective (*n* = 1702). Multiple selections of pre-existing conditions, complications, or medications were possible in the questionnaire. There was no constraint in answering each questionnaire item. The radiologist in charge discussed missing critical questionnaire items in the personal informed consent discussion and added documentation manually in the Portable Document Format (PDF)

*All three patients were at higher age (> 60 years) and pregnancy was ruled out in the personal informed consent discussion

Most patients had previously received a CT examination (87%) and had a prior iodine-containing contrast agent injection (86%). More than half of the patients suffered from malignant diseases (66.9%), and about half were currently receiving or had completed chemotherapy (51.6%). Several patients reported anticoagulant medication (24.6%), which could be of interest in the case of interventional procedures. Metal implants, potentially limiting CT image quality and demanding additive metal artifact reduction techniques, were reported in a considerable portion (19.7%). Details of general patient history are available in Table 3.

None of the mobile tablet devices were lost, stolen, or broken during the study period. The patients did not claim any system instabilities or crashes during the processing in the waiting room. We experienced no disturbances in the clinical workflow by the disinfection or the return of the tablets from the preparation room to the front desk, especially compared to the conventional paper-based workflow.

### Physician client

Red flags were present in 61.3% (*n* = 1043) of all included patients. The most prevalent red flags were thyroid diseases (23.8%). However, the critical contraindication for iodine-containing contrast-agent injection thyroid hyperfunction was relatively rare (2.0%). Also, kidney diseases were often highlighted as red flags (12.5%), but the prevalence of limiting reduced kidney function (3.6%) or hemodialysis (0.6%), where contrast agent injection may be contraindicated or dedicated patient preparation has to be provided, was relatively low. Only a tenth of all patients reported diabetes medications (9.6%), and 5.5% of the diabetes patients reported metformin medication in their anamnesis, accounting for only 0.5% of all included patients in this study. One patient reported simultaneously reduced kidney function (0.06%). Patients with metformin medication and reduced kidney function must receive intensive examination preparation due to the potential risk of lactate acidosis. A history of severe allergic reactions against iodine-containing contrast agents was reported in only a few cases (0.3%). Mild to moderate allergic reactions had a higher prevalence (4.9%). Consequently, every twentieth patient required a more detailed evaluation of potential allergic reactions or adaptation of the examination protocol. Overall, a cumulative 13.7% of patients had a potential critical patient history requiring dedicated CT preparation: 5.8% prior complications after contrast-agent application, 0.5% metformin-containing drugs, 2.0 thyroid hyperfunction, 0.8% thyroid cancer, 0.4% hot thyroid nodule, 3.6% reduced kidney function, 0.6% hemodialysis. A substantial proportion of patients also reported general claustrophobia (9.7%). Details of red flag items are available in Table 4.

**Fig. 3:**
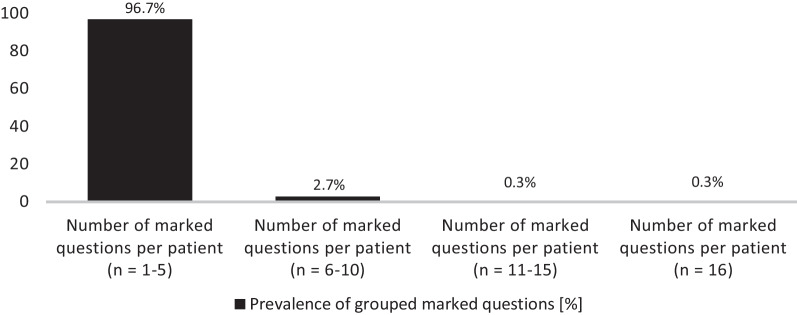
Grouped prevalence [%] of marked questions (total marked questions in the study cohort *n* = 370/1702 = 21.7%). The figure illustrates that patients with marked questions mostly had *n* = 1–5 inquiries (*n* = 358; 96.7%). Only a minority of patients had 6–10 inquiries (*n* = 10; 2.7%), 11–15 inquiries (*n* = 1; 0.3%), or 16 inquiries (*n* = 1; 0.3%)

Marked questions were present in 21.7% (*n* = 370; range 1–16 questions) of all study participants. Patients who marked one or more questions were significantly older (63.9 (IQR 57–72) years) than patients without inquiries (59.5 (IQR 52–68) years; *p* < 0.001). Most patients (96.7%) marked 1–5 questions and were predominantly male (56.6%). The minority of patients marked more than 5 questions (details are illustrated in Fig. 3).

Interaction with different filters in the physician client was fast and intuitive. However, the latency of up to 10 s to generate the PDF file affected the subjective performance experience. The signed D-IPC documents were successfully archived in our ePACS. We initially experienced some confusion retrieving the PDF via the RIS system because the staff had to adapt to the new workflow. However, it was overall fast and accessible with only one click.

### Sustainability

The study helped avoid using 3404 sheets of paper and correspondingly saved 14.6–16.1 kg CO_2eq_ paper-related GHG emissions. However, estimated CO_2eq_ emissions for the 12 tablet devices (1312 kg CO_2eq_) were 80–90 times higher.

### Cost–benefit analysis

The CBA (Table 5) shows higher initial costs for D-IPC due to hardware acquisitions and IT service costs (1st year 29145.2 vs. 17971.5 Euro; + 62.2%). In the following years (2nd to 5th year) the yearly estimated operational costs for D-IPC are less (14965.2 vs. 15971.5 Euro; − 6.7%). This is mainly caused by omitted operational costs for manual document archiving. However, the yearly operational costs for the IT service are still high (4620 vs. 1049 Euro; + 340%). The yearly hardware energy costs are moderate (63.6 Euro). Also, the yearly paper costs (40.32 Euro) and printing costs (282.24 Euro) for 4032 pages are low in comparison to the personnel operational costs. Additionally, we identified three major performance features for D-IPC (1: high availability and readability of patient information on each workstation, 2: pre-filled questionnaire forms for recurrent CT examinations, 3: structured access and evaluation of red flag questions for potential contraindications), and one major performance feature for C-IPC (1: ease of use for patients with reduced digital literacy), which help improving quality of patient care. The total costs over a 5-years project duration are slightly higher for D-IPC (89006 vs. 81857.3 Euro; + 8.7%).

**Table 5:** Cost–benefit analysis for digitized versus conventional informed patient consent (D-IPC vs C-IPC)

Total count of IPC forms = 2016	Years									
	1		2		3		4		5	
	D-IPC	C-IPC	D-IPC	C-IPC	D-IPC	C-IPC	D-IPC	C-IPC	D-IPC	C-IPC
*A. Personnel—Operational Costs (€)*										
A-1. IPC preparation and patient assignment by technicians or support staff	4233.6	4233.6	4233.6	4233.6	4233.6	4233.6	4233.6	4233.6	4233.6	4233.6
A-2. Manually archiving IPC forms by technicians or support staff	0	4233.6	0	4233.6	0	4233.6	0	4233.6	0	4233.6
A-3. Estimated time effort of physicians to complete IPC process	6048	6048	6048	6048	6048	6048	6048	6048	6048	6048
A-4. Costs of wrong archival storage and retrieval efforts	0	84.7	0	84.7	0	84.7	0	84.7	0	84.7
*B. IT service—operational costs (€)*										
B-1. Installation process (server software)	7500	2000	0	0	0	0	0	0	0	0
B-2. Installation process (tablet device)	1080	0	0	0	0	0	0	0	0	0
B-3. Maintenance and Support Services	3168	0	3168	0	3168	0	3168	0	3168	0
B-4. Network/Hosting Services	243	243	243	243	243	243	243	243	243	243
B-5. Costs per analog questionnaire (50 cents)	0	1008		1008	0	1008	0	1008	0	1008
B-6. Cost per digital questionnaire (40 cents) and digital signature (20 cents)	1209	0	1209	0	1209	0	1209	0	1209	0
*C. Hardware—operational costs (€)*										
C-1. Physician tablet (n = 2)	1600	0	0	0	0	0	0	0	0	0
C-2. Patients tablet (*n* = 10)	4000	0	0	0	0	0	0	0	0	0
C-3. Energy costs tablets	63.6		63.6		63.6		63.6	0	63.6	0
C-4. Paper costs	0	40.32	0	40.32	0	40.32	0	40.32	0	40.32
C-5. Printing costs	0	282.24	0	282.24	0	282.24	0	282.24	0	282.24
*D. others—operational costs (€)*										
D-1. Disposal of informed patient consent documentation (after 30 years)	0	n/a	0	n/a	0	n/a	0	n/a	0	n/a
*E. Quality improvement of patient care*										
E-1. High availability and readability of patient information in each workplace	++	−	++	−	++	−	++	−	++	−
E-2. Pre-filled questionnaire forms for recurrent CT examinations	++	−	++	−	++	−	++	−	++	−
E-3. Structured access and evaluation to red flag questions for potential contraindications	++	−	++	−	++	−	++	−	++	−
E-4. Ease of use for patients with reduced digital literacy	−	+	−	+	−	+	−	+	−	+
Total costs per annum (€)	29145.2	17971.5	14965.2	15971.5	14965.2	15971.5	14965.2	15971.5	14965.2	15971.5

Total costs (€) (5 years)	
Digitized informed patient consent	89006 (+ 8.7%)
Conventional informed patient consent	81857.3

### Database comparison

Patient history and clinical context information were completely missing in four (0.2%) patients. For 1276 (75%) patients, only the clinical question for the CT examination was fully provided. However, the clinical context (e.g., the kind of malignancy, current symptoms, recent changes in therapy) was incomplete. Consequently, missing patient information had to be retrieved from D-IPC, RIS, and the hospital information systems. Some severe discrepancies between the documented red flags by D-IPC and C-PR were found (Table 6). None of the D-IPC cases with a history of thyroid hyperfunction was documented in the C-PR (34 vs 0 cases). Also, for reduced kidney function (65 vs 2 cases), infectious diseases (51 vs 0 cases), hemodialysis (12 vs 1 case), and claustrophobia (214 vs 4 cases), large discrepancies between the documentation with D-IPC versus C-PR were apparent. The discrepancy was less for allergies against iodine-containing contrast agents (70 vs 40 cases).

**Table 6:** Comparison of red-flag prevalence documented with entirely digitized, tablet-based informed patient consent (D-IPC) versus the data documented in the conventional patient records (C-PR)

Questionnaire item	D-IPC (*n*)	C-PR (*n*)
Allergies against iodine-containing contrast agents	4.9% (84)	2.3% (40)
Reduced kidney function	3.6% (62)	0.1% (2)
Hemodialysis	0.6% (11)	0.05% (1)
Thyroid hyperfunction	2.0 (34)	0% (0)
Infectious disease	2.8% (28)	0% (0)
Claustrophobia	9.7% (166)	0% (0)

## Discussion

Our study proves that D-IPC is feasible before contrast-enhanced CT with a more than 80% success rate. The primary limiting factor is patient age, even when the condensed questionnaire focusing on the CT examination is used. Red flags are highly prevalent in this study (61%). We focused on being sensitive to any potential contraindications. Therefore, the red flag items are mostly defined as general organ issues (e.g., thyroid disease, kidney disease) and not specific pathologies (e.g., thyroid hyperfunction, renal insufficiency). This leads to high a red flag prevalence and potentially false-positive findings. However, patients may have heterogeneous and sometimes suboptimal health literacy, which could cause false-negative answers. Finally, it is obligate to the radiologist to clarify all red flag issues. Digitized and structured red flags can focus and personalize the physician–patient discussion, help optimize the examination protocol, complete and structure information about critical patient issues. Additionally, we see in the clinical routine that improved data availability on each workstation, quick document retrieval, optimal readability, and pre-filled questionnaire item for recurrent examinations are major benefits for our patients and the hospital staff, which outweighs the higher initial costs of D-IPC compared to C-IPC. Moreover, we found a critical discrepancy in the red flag documentation rate between D-IPC and C-PR. We believe D-IPC documentation with the information provided directly from the patients is more accurate and reliable than C-PR. Nevertheless, we experienced that C-PR is often incomplete because verbally transmitted information or information on handwritten notes provided by the referring physicians are incompletely archived due to time pressure and several workflow interruptions. This may contribute to a bias of unknown extent for the red flag prevalence in C-IPC.

Overall, 15.5% of the patients answered the questionnaire incompletely. This portion is less than the 22.1% previously reported for conventional paper-based informed consent by Vogele et al. [9] who reported 18.8% incomplete and 3.3% empty questionnaires. Patients who could not complete the D-IPC before CT examinations were significantly older than the subgroup who successfully finalized it. This limitation copies the findings from our previous study where only 7% of 317 patients were excluded due to refusal, interruption, or incomplete digitized anamnesis before CT [16]. Therefore, the length of D-IPC seems not to influence the success rate.

The positive feedback from the clinical routine is in line with the performance of other systems in the literature. For example, Abujarad et al. [19] reported in a randomized controlled trial that participants had higher satisfaction, higher ability to complete the consent independently, and shorter perceived time to complete the consent process using a different D-IPC system. Hess et al. showed that most primary care patients (84%) had no difficulties using a tablet computer-based questionnaire with routine screening content. However, they also report that increasing age was a predictor of increased difficulty completing the questionnaire [20].

Considering the general patient history, information about prior contrast-agent applications (7.6%) and malignant disease (6.8%) was most frequently missing in the D-IPC. The most frequently unanswered red flag questions were kidney disease (4.8%) and thyroid disease (3.9%). We assume the answers to such questions are partly unknown to the patients. This finding also underlines the need for better synchronization of the hospital information systems with IPC systems in general and radiological systems in this particular situation.

For the three-fourths of patients with incomplete information about patient history in the RIS, a time-consuming search in the hospital information system, review of the paper-based patient records, or phone calls are potentially required to clarify the CT indication. D-IPC could help improve data availability and, consequently, CT protocol selection and report quality in these cases. A cumulative 13.7% of all patients had a potential critical patient history for CT with contrast-agent application. An examination without dedicated preparation or protocol adaptations could unnecessarily harm such patients. Without structured D-IPC, patient safety is more and more at risk because increasing amounts of patient information with moderate relevance may distract from the most critical issues. Especially in the follow-up setting, pre-filled digitized questionnaires are promising to facilitate the CT preparation process.

Marked questions were recorded at a reasonable frequency (21.7%), and most patients had only a few inquiries. This functionality helped the radiologist to personalize the patient–physician interaction to the background knowledge of our patients. However, the significant older age of patients with marked questions indicates that older patients may not be familiar with digital devices or have the same health literacy as younger patients.

Lastly, the saved GHG emissions due to paperless D-IPC are comparable to a 150–160 km car ride, which complies with recent regulations in the European Union allowing a maximum emission of 95 g CO_2_/kilometer [21]. Some studies evaluated the GHG savings of digital information services and provided conflicting results, depending on the number of readers per paper sheet or book and the energy efficiency of the electronic reading devices (e.g., tablet computer, e-ink tablet, or personal computer [15, 22]). However, the estimated CO_2eq_ for the tablet production provided by the vendor is 80–90 times higher in this study. Consequently, the greenhouse gas emissions of paper production are only a minor factor.

Additionally, the CBA illustrates that D-IPC is an innovative solution with benefits for structured and personalized patient care. However, this innovation is accompanied by slightly higher total operational costs. Consequently, future developments should focus on cost-efficient IT services and hardware solutions.

## Limitations

First, it is unknown how accurately patients answer the questionnaire. For example, three female patients specified pregnancy, but all were at higher age (> 60 years), and pregnancy was ruled out in the patient–physician interaction. However, the same problem is present for C-IPC as well. In both scenarios, the signature affirms the validity of health information. Second, D-IPC makes sufficient data security and power supply mandatory. Third, a modern hospital infrastructure with optimal WLAN performance and Department of Informatics support is necessary. Fourth, the evaluated software did not provide timestamps for measuring D-IPC duration. Also, we could not measure the exact duration of conventional C-IPC. Therefore, an exact comparison of time effort between both methods is impossible. Indeed, we did not observe changes of CT examination numbers during this clinical routine study, which indicates comparable total time effort. Fifth, in the retrospective database evaluation, we could only count the total number of marked questions for each patient, but we had no insight into which questions were marked. Sixth, we could not register the number of patients who refused D-IPC before the assignment. Such patients were never digitally registered in the D-IPC software. Seventh, the CBA is based on calculations highly influenced by local prices (e.g., IT services, national wage contracts). Therefore, international institutions should especially consider the local personnel's operational costs. Lastly, future developments will indicate, how many institutions and radiology sites are able and willing to pay the additional charges accompanied by D-IPC. Especially, when many international health care systems are under economic pressure.

## Conclusion

D-IPC is feasible for the majority of patients but older patients have more difficulty coping with D-IPC. Structured red flags and marked questions supports efficient, personalized patient–physician interaction. Moreover, C-PR documentation covers only a minority of the red flags recorded with D-IPC. D-IPC is slightly more expensive but has the potential to improve patient safety significantly. Furthermore, GHG emissions are much higher for D-IPC due to tablet production. Future developments of D-IPC should approach these shortcomings.

## Data Availability

The datasets generated and analyzed during this study are available from the corresponding author on reasonable request.

## Abbreviations

.XML: Extensible markup language file
CBA: Cost–benefit analysis
CO_2eq_: Carbon dioxide equivalent
C-PR: Conventional paper-based patient records
D-IPC: Digitized informed patient consent
ePACS: Enterprise picture archiving and communicating system
ESUR: European Society of Urogenital Radiology
GHG: Greenhouse gas
IPC: Informed patient consent
IQR: Interquartile range
PDF: Portable document format
RIS: Radiology information system
